# Understanding the Role of Environmental Transmission on COVID-19 Herd Immunity and Invasion Potential

**DOI:** 10.1007/s11538-022-01070-y

**Published:** 2022-09-10

**Authors:** M.A Masud, Md. Hamidul Islam, Byul Nim Kim

**Affiliations:** 1 Natural Product Informatics Research Center, Korea Institute of Science and Technology, Gangneung, 25451 South Korea; 2 Department of Applied Mathematics, University of Rajshahi, Rajshahi, 6205 Bangladesh; 3 Institute for Mathematical Convergence, Kyungpook National University, Daegu, 41566 South Korea

**Keywords:** COVID-19, Vaccination, Indirect transmission, Mathematical modeling, Identifiability

## Abstract

COVID-19 is caused by the SARS-CoV-2 virus, which is mainly transmitted directly between humans. However, it is observed that this disease can also be transmitted through an indirect route via environmental fomites. The development of appropriate and effective vaccines has allowed us to target and anticipate herd immunity. Understanding of the transmission dynamics and the persistence of the virus on environmental fomites and their resistive role on indirect transmission of the virus is an important scientific and public health challenge because it is essential to consider all possible transmission routes and route specific transmission strength to accurately quantify the herd immunity threshold. In this paper, we present a mathematical model that considers both direct and indirect transmission modes. Our analysis focuses on establishing the disease invasion threshold, investigating its sensitivity to both transmission routes and isolate route-specific transmission rate. Using the tau-leap algorithm, we perform a stochastic model simulation to address the invasion potential of both transmission routes. Our analysis shows that direct transmission has a higher invasion potential than that of the indirect transmission. As a proof of this concept, we fitted our model with early epidemic data from several countries to uniquely estimate the reproduction numbers associated with direct and indirect transmission upon confirming the identifiability of the parameters. As the indirect transmission possess lower invasion potential than direct transmission, proper estimation and necessary steps toward mitigating it would help reduce vaccination requirement.

## Introduction

Coronaviruses are enveloped RNA viruses that use mammals and birds as hosts and have the ability to cause various types of respiratory symptoms (Wardeh et al. 2021; Zhu et al. 2020; Kim and Lee 2020). Two distinguished strains of this virus, namely SARS-CoV and MERS-CoV, have caused several epidemic outbreaks during the last two decades at several places around the world (Zhu et al. 2020). The ubiquity of this virus along with its large genetic diversity and increasing animal–human interactions has amplified the likelihood of the emergence of a coronavirus infection (Huang and Wang 2021). The most recent outbreak of the virus was caused by the novel strain SARS-CoV-2 that led to the recent pandemic of the coronavirus disease 2019 (COVID-19).

In the last two years, significant improvement has been done in understanding the transmission routes and pathways of COVID-19 (Azuma et al. 2020; Rothe et al. 2020; Yu and Yang 2020; Morawska et al. 2020; Pitol and Julian 2021; Castaño et al. 2021). The onset of this disease is usually characterized by symptoms like fever, cough, and sore throat, and in some cases, the severity of the disease leads to shortness of breath. Virus particles discharged through nostrils and mouth during breathing, talking, sneezing, and/or coughing may transmit the disease to other host. COVID-19 patients may spread the disease at least 1–3 days before the onset of their symptoms (Wormser 2020). Furthermore, in many cases (17.8% Mizumoto et al. 2020, 30.8% Nishiura et al. 2020), it has been shown that patients tend to be asymptomatic or simply develop very mild symptoms throughout the entire infectious period. Consequently, patients who are infectious and transmit the disease may go unnoticed, which can be a key driver that undermines any efforts to contain the disease (Bai et al. 2020; Rothe et al. 2020). Another potential driver for transmission could be the prolonged sustenance of the virus on environmental fomites (Vardoulakis et al. 2020; Azuma et al. 2020; Pitol and Julian 2021). In experimental setup, SARS-CoV-2 was found stable on plastic and stainless steel up to 72 h (van Doremalen et al. 2020). On plastic and human skin surfaces, variants of SARS-CoV-2 maintained infectivity for several hours (Hirose et al. 2020, 2022). Gidari et al. reported infectious existence of this virus on plastic and glass for more than 120 h and on stainless steel for more than 72 h (Gidari et al. 2021). Infectious virus was detected even after 7 days on a sample of surgical masks (Chin et al. 2020). SARS-CoV-2 survival for up to 1, 5, and 10 days was reported on fake fur, plastic, and mink fur, respectively (Brown et al. 2021). In artificial saliva, it was found stable for at least 90 min (Smither et al. 2020). Live SARS-CoV-2 RNA was detected on $8.3\%$ of the high-touch surfaces in the public locations during a COVID-19 outbreak in Massachusetts (Harvey et al. 2021). The above literature suggests that an additional key driver for COVID-19 outbreak could be the prolonged sustenance of the virus on environmental fomites. However, the effectiveness of surface disinfection is highly dependent on the prevalence and the frequency of contact as well as environmental conditions (Gidari et al. 2021; Pottage et al. 2021; Wilson et al. 2021). For instance, approximately $30\%$ of disease transmissions on the Diamond Princess cruise ship were reported to be related to fomite-mediated transmission (Azimi et al. 2021), whereas in China this percentage is reported to be 45–62% (Yang and Wang 2021). In hospital setting, $27\%$ of the environmental surfaces were reported to contain SARS-CoV-2 RNA even though disinfectant were sprayed twice (Kim et al. 2020). Further details pertaining to the deposition, survival, and transmission of the virus can be accessed in Leung (2021), Castaño et al. (2021), Aydogdu et al. (2021), and Gonçalves et al. (2021). The environmental transmission has also been observed to play a critical role in the persistence and inter annual epidemics for other communicable diseases (Vergara-Castaneda et al. 2012; Lopman et al. 2012; McKinney et al. 2006; Breban et al. 2009; Al-Tawfiq and Memish 2016).

Environmental route of transmission has been modeled mathematically for several infectious diseases and was proved to hold important implications for disease control (Eisenberg et al. 2005; Zhao et al. 2012). For instance, environmental transmission modulates the periodicity in avian influenza outbreak (Breban et al. 2009; Rohani et al. 2009; Wang et al. 2012). It is also associated with spatial diffusion of avian influenza (Li et al. 2019). Recently, several mathematical models have been proposed regarding fomite-mediated transmission of COVID-19 (Yang and Wang 2020; Stutt et al. 2020; Yang and Wang 2021; Wijaya et al. 2021; Rwezaura et al. 2021). However, the role of fomite-mediated transmission in crucial public health issues, such as herd immunity, has yet to be substantially explored. Moreover, it would be of interest to evaluate which transmission pathway has a higher invasion potential. In this study, we have classified the transmission routes into two types–direct and indirect. Direct transmission refers to transmission of the infection that comes directly from an infectious to a susceptible individual. In contrast, indirect transmission refers to the deposition of the virus particles by an infectious individual on environmental fomites followed by inoculation of the virus by a susceptible individual who, in turn, becomes infectious.

The remaining part of the paper is structured as follows. We initially present the mathematical model in Sect. 2. We then analyze our model to establish the disease invasion threshold and investigate its sensitivity to both transmission routes in Sect. 3.1. Section 3.2 presents the stochastic simulation wherein we investigate the invasion potential of both transmission routes. Consequently, we fit our model to the early epidemic data obtained from several countries, along with identifiability analysis to quantify route-specific transmission strength (in Sect. 3.3), thereby measuring the vaccination requirement according to initial outbreak data for the acquisition of herd immunity, which is presented in Sect. 3.4. Finally, we discuss and summarize our findings in Sect. 4.

**Fig. 1:**
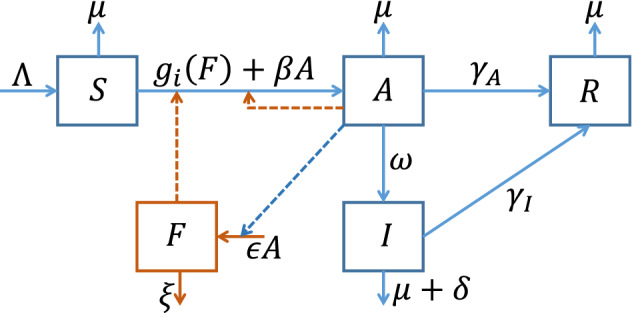
Flowchart. The squares represent the compartments, solid lines show the flow between the compartments, and dotted line demonstrates the inducing effect of the compartment on the respective flow rate (color figure online)

## Epidemic Model

We divided the human host into four different compartments: susceptible (*S*), infectious (*A*), confirmed infected (*I*), and recovered (*R*). Furthermore, *F* represents viruses on environmental carriers or fomites (Fig. 1). Susceptible individuals are the ones who can contract the disease following exposure to the virus. Once a susceptible person is exposed to the virus through direct or indirect contact with an infectious agent, they may either become infectious and express symptoms after a latency period or may not express symptoms albeit transmit the disease. Depending on symptom expression, public health guidelines and the capacity of public health authority to isolate infectious individuals, the infectious individuals may be confirmed/identified as infected, or may remain unnoticed and remain infectious. For simplicity, we assume the confirmed infected individuals no longer transmit the disease. An infected individual may remain infectious throughout his/her whole infectious lifetime and pass through $S \xrightarrow {} A \xrightarrow {} R$ pathway; or an infected individual may remain infectious in first few days until s/he becomes confirmed at some point of his/her infectious lifetime and pass through $S\xrightarrow {}A\xrightarrow {}I\xrightarrow {}R$ pathway.

When infectious individuals talk loudly, cough, or sneeze, numerous virus particles exit from their respiratory organs and can be deposited on surfaces in the environment, where they can survive for a long time (van Doremalen et al. 2020; Hirose et al. 2020, 2022; Gidari et al. 2021; Chin et al. 2020; Brown et al. 2021; Smither et al. 2020; Harvey et al. 2021) and be carried away by a new host afterward. Virus particles deposited on environmental fomites belong to the *F* compartment. The rate at which asymptomatic individuals *deposit* the virus on fomites is $\epsilon$ and the viral particles *decay* naturally at a rate of $\xi$. We assume that the viral population is large enough to describe the viral population dynamics by the following two processes: *deposit* and *decay*. In addition, we consider that the probability of infection from a virus picked up from the environmental fomites is a function of daily viral exposure to the environmental fomites. Precisely, we assume that a constant fraction $\rho$ of virions (*F*) is picked up by each susceptible individual per day and may cause infection with a probability of $g(F_d)$, where $F_d=\rho F$, which reflects the daily pick up rate. We consider the following two types of functional forms for *g*, which are written as a function of *F* only for simplicity, as $\rho$ is assumed constant.

Case I $g_1(F)= \pi \rho F = \alpha F$ (Li et al. 2009).
Case II $g_2(F)= 1-e^{-\pi \rho F} = 1-e^{-\alpha F}$ (Watanabe et al. 2010; Breban et al. 2009).

where $\alpha = \pi \rho$. Under these assumptions, we obtain the following system of differential equations

1 $$\begin{aligned} \frac{\mathrm{d}S(t)}{\mathrm{d}t}= & {} \Lambda - \beta A S - S g_i(F) - \mu S \nonumber \\ \frac{\mathrm{d}A(t)}{\mathrm{d}t}= & {} \beta A S + S g_i(F) - (\omega + \gamma _A + \mu ) A \nonumber \\ \frac{\mathrm{d}I(t)}{\mathrm{d}t}= & {} \omega A - ( \gamma _I + \mu + \delta ) I \nonumber \\ \frac{\mathrm{d}R(t)}{\mathrm{d}t}= & {} \gamma _A A + \gamma _I I - \mu R\nonumber \\ \frac{\mathrm{d}F(t)}{\mathrm{d}t}= & {} \epsilon A - \xi F \end{aligned}$$

## Results

### Invasion Threshold

The invasion threshold of the disease is determined by the existence and stability of the equilibria.

#### Proposition 1

The model has a unique disease-free equilibrium (DFE), $\mathcal {E}_0$. In addition, it has an endemic equilibrium (EE), $\mathcal {E}$ which exists for $R_0>1$.

The basic reproduction number, $R_0$, is a crucial threshold for characterizing the dynamics of an outbreak. It refers to the average number of secondary infections caused by the introduction of one infectious individual in a completely susceptible population. Here, we designate *A* and *F* as the diseased class. The DFE is given by $\mathcal {E}_0=\left( \frac{\Lambda }{\mu }, 0, 0, 0, 0\right)$.

The next generation matrix at $\mathcal {E}_0$ is given by (Please refer to “Appendix A” for details)

2 $$\begin{aligned} K = [K_{i,j}] = \mathfrak {FV}^{-1} = \left( \begin{array}{cc} \frac{\Lambda \beta }{\mu k_A} &{} \frac{\Lambda f_i'(0)}{\mu \xi } \\ \frac{\epsilon }{k_A} &{} 0\\ \end{array} \right) \end{aligned}$$

where $$k_A = \omega + \gamma _A + \mu $$ and $$k_I = \gamma _I + \mu + \delta $$.

$K_{k,j}$ provides the expected number of secondary infections in class *k* produced by a single incident in class *j*. Recent studies (Yang and Wang 2020; Stutt et al. 2020; Yang and Wang 2021; Wijaya et al. 2021), $K_{2,1}$ is considered 0 without considering the environmental fomites (*F*) as infectious. In this study, we consider both *A* and *F* as infectious compartments, and we interpret $K_{1,1}$ as the number of new secondary infectious individuals caused by one infectious individual during his/her entire infectious period, $K_{2,1}$ as the number of virus particles spread by an infectious individual throughout his/her entire infectious period, and $K_{1,2}$ as the number of new infected individual caused by one virus particle in the environment throughout its entire active period. The spectral radius of *K* is the basic reproduction number $R_0$, which is given below

$$\begin{aligned} R_0 = \rho (K) = \frac{1}{2}\left( \frac{\Lambda \beta }{\mu k_A}+ \sqrt{\left( \frac{\Lambda \beta }{\mu k_A}\right) ^2+4\frac{\Lambda \epsilon \rho \pi }{\mu \xi k_A }}\right) \end{aligned}$$

The basic reproduction number can be rearranged and expressed in the following form:

$$\begin{aligned} R_0 = \frac{1}{2}\left( R_{0H}+ \sqrt{R_{0H}^2+4R_{0F}}\right) \end{aligned}$$

where $$R_{0H} = \frac{\Lambda \beta }{\mu k_A}$$, $$R_{0F}= \frac{\Lambda \rho \epsilon \pi }{\mu \xi k_A }$$.

Here, we can highlight the role of direct and indirect transmission. At $\mathcal {E}_0$, an asymptomatic infectious individual transmits disease to $\frac{\Lambda }{\mu }\beta$ individuals per day and the total infectious period is $\frac{1}{k_A}$ days. Therefore, $R_{0H}=\frac{\Lambda }{\mu }\beta \frac{1}{k_A}$ is the expected number of new infections generated from an infected individual throughout his/her entire infectious period. In contrast, infectious individuals deposit virus particles on environmental fomites at a rate of $\epsilon$. The total virus particles deposited in the environment throughout the entire infectious period of a single infected individual is $\frac{\epsilon }{k_A}$. Each virus particle can subsequently infect a person with a probability of $\alpha$ in Case I and $1-e^{-\alpha }\approx \alpha$ in Case II per day. A virus particle can survive on environmental fomites for an expected duration of $\frac{1}{\xi }$ days. Hence, the expected number of infected individuals caused by a single virus particle in a completely susceptible environment is $\frac{\Lambda }{\mu }\frac{1}{\xi }\alpha$. Therefore, $R_{0F} = \frac{\Lambda \epsilon \pi \rho }{\mu \xi k_A}$ quantifies the expected number of secondary infections as a result of indirect transmission.

**Fig. 2:**
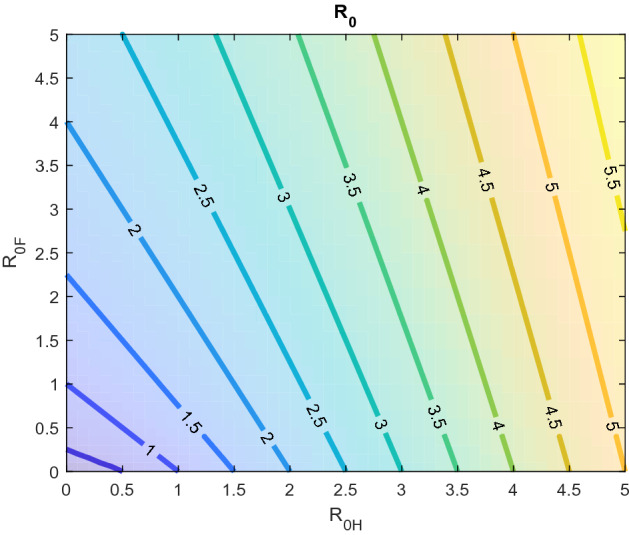
Role of direct $(R_0H)$ and indirect $(R_0F)$ transmission in outbreak (color figure online)

A comparison of the role of direct and indirect transmission is portrayed in Fig. 2, which shows that $R_0$ increases linearly with $R_{0H}$. In contrast, $R_0$ increases faster than linear with $R_{0F}$ until $R_0<1$. When $R_0>1$, the impact of indirect transmission diminishes as $R_0$ increases, i.e., indirect transmission plays a crucial role if $R_0$ is near 1.

#### Theorem 1

The DFE is locally asymptotically stable for $R_0<1$ and unstable for $R_0>1$.

Please refer to “Appendix B” for detailed proof. The EE is given by

$$\begin{aligned} \mathcal {E_*}=(S_*, A_*, I_*, R_*, F_*) \end{aligned}$$

where $S_*= \frac{k_A}{\beta +g_i(F) /A_*}$, $I^* = \frac{\omega A_*}{k_I}$, $R_*=\frac{\gamma _A A_* + \gamma _I I_*}{\mu }$ and $F_* = \frac{\epsilon A_*}{\xi }$. For Case I, $A_*=\frac{\mu }{\beta }\frac{R_{0H}+R_{0F}-1}{1+\frac{R_{0F}}{R_{0H}}}$ and for Case II, $A_*$ is given by

$$\begin{aligned} (R_{0H}+1)\mu -\beta A_* = \left( 1-\frac{\Lambda }{A_* k_A}\right) \left( 1-e^{-\frac{\alpha \epsilon A_*}{\xi }} \right) \end{aligned}$$

It is clear that $(1-e^-\frac{\alpha \epsilon A_*}{\xi })\in [0, 1]$, otherwise the system will be unbounded below. Hence, we obtain

$$\begin{aligned} A_*^2+\frac{1-(R_{0H}+1)\mu }{\beta }A_*-\frac{\Lambda }{\beta K_A}\le 0 \end{aligned}$$

which has, at most, one positive solution for $A_*$ as $\frac{1-(R_{0H}+1)\mu }{\beta }A_*\ge 0$ since $\mu<<1$. For Case I, the expression of $A_*$ demonstrates that the EE exists for $R_{0H}+R_{0F}\ge 1$. However, it is cumbersome to deduce the condition for the existence of $A_*$ in Case II. Therefore, we chose to use the center manifold theorem (Theorem 4.1 in Castillo-Chavez and Song 2004), which could help us characterize the existence and nature of the EE near $R_0=1$ using the Jacobian at the DFE for both the cases simultaneously.

#### Theorem 2

The model exhibits forward bifurcation at $R_0=1$.

Please refer to “Appendix C” for detailed proof. The above analysis nominates $R_0=1$ as the disease invasion threshold. The expression of $R_0$ clarifies the role of both direct and indirect transmissions. $R_0$ can be used to measure the control efforts required to mitigate or stop the spread of the disease. However, if the infection is carried by more than one types of host, the use of $R_0$ leads to a distinct underestimation of the requirements (Bani-Yaghoub et al. 2012; Pauline 2017). In such cases, the type reproduction number provides a significantly more accurate estimation of the required control efforts (Roberts and Heesterbeek 2003; Heesterbeek and Roberts 2007).

#### Type Reproduction Number

It is essential to gain a clear understanding of the explicit role of human carriers (direct transmission) and environmental carriers (indirect transmission) in spreading the virus so as to decide feasible and effective control strategies. The basic reproduction number properly defines the invasion threshold, but this number cannot distinguish the pathway-specific transmission. In this section, we use the concept of type reproduction number (Roberts and Heesterbeek 2003; Heesterbeek and Roberts 2007) to investigate the pathway-specific transmission. Type reproduction number for host, $T_H$, refers to the expected number of infectious individuals caused by one infectious individual in a completely susceptible environment, either by direct or indirect transmission. Following the notation in Roberts and Heesterbeek (2003), let $I_5$ be the $5\times 5$ identity matrix, $P_H=[ph_{ij}]$ be the projection matrix defined by, $ph_{11}=1$, and $ph_{ij}=0$ when $i\ne 1$ or $j\ne 1$. $E_H$ is the unit column matrix with its first element equal to 1. Then,

$$\begin{aligned} T_H = E_H'K(I_5-(I_5-P_H)K)^{-1}E_H= R_{0H}+R_{0F} \end{aligned}$$

and we have the following properties (Heesterbeek and Roberts 2007).

- $$T_H>1$$ iff $$R_0>1$$.
- Transmission will be terminated over time if $T_H$ can be reduced by a factor of $v_H\ge 1-\frac{1}{T_H}$. This reduction can be achieved by means of vaccinating susceptible individuals (as this will reduce the number of available susceptible individuals) or by quarantining infectious individuals (as this will reduce their infectious period).
- Finally, $\rho ((I-P)K)=0$, which indicates that fomites do not act as a reservoir.

**Fig. 3:**
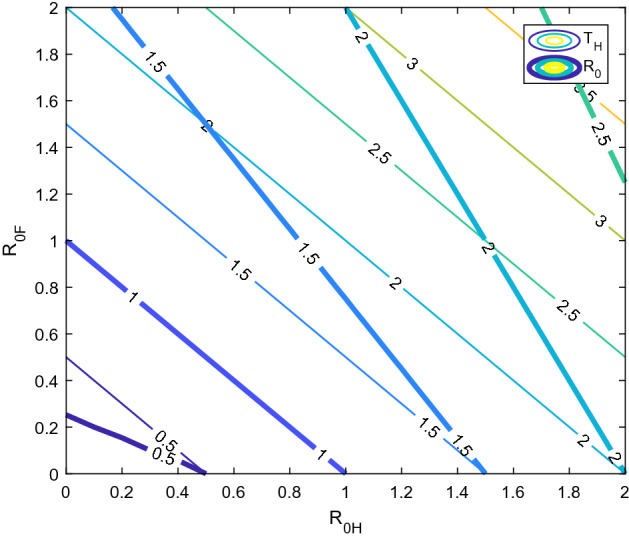
Comparison of the $R_0$ and $T_H$. For the same combination of $R_{0H}$ and $R_{0F}$, we compared the value of $R_0$ (thick lines) and $T_H$ (thin lines) using a contour plot (color figure online)

Therefore, the invasion threshold can be refined in terms of the type reproduction number as $T_H=1$. Furthermore, although the expression for $R_0$ is difficult to interpret from the biological point of view, the expression for $T_H$ can be easily understood as the total number of the expected secondary infected individuals as a result of both the direct and indirect transmissions caused by one infectious individual in a completely susceptible environment. Figure 3 shows a comparison between $R_0$ and $T_H$ illustrating that they both coincide at 1, but $R_0>T_H$ below 1 and $R_0<T_H$ above 1, which may also be confirmed by using simple algebra. Rephrasing the invasion threshold not only allows us to provide a biological interpretation but also leads us to differentiate the pathway-specific transmission strength and infer the relative requirement of the subsequent control measures (Heesterbeek and Roberts 2007). If the value of $T_H$ is known, we can estimate the vaccination requirement. Further, if we can distinguish $R_{0H}$ and $R_{0F}$, i.e., isolate pathway-specific transmission strength, we will be able to estimate required strictness in maintaining quarantine measures, and requirement of cleanliness and maintaining personal hygiene to an appropriate degree.

### Stochasticity in Invasion

**Fig. 4:**
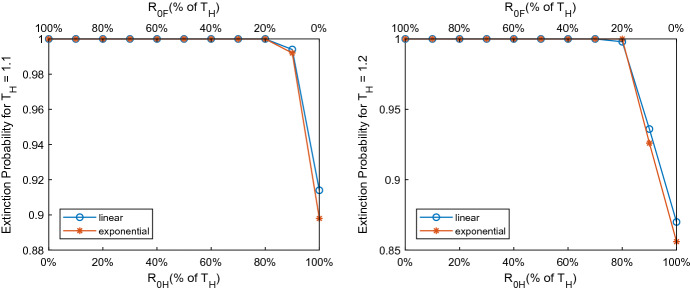
Extinction probability. Approximate extinction probabilities for different combinations of $R_{0H}$ and $R_{0F}$ corresponding to the same $T_H$ have been shown. The figure on the left is for $0\ge R_{0F},R_{0H}\ge 1.1$, whereas the figure on the right is for $0\ge R_{0F},R_{0H}\ge 1.2$ (color figure online)

The expression of invasion threshold, $T_H$ demonstrates that it is equally sensitive to $R_{0H}$ and $R_{0F}$. For $T_H$ slightly greater than 1, there might exist a nonzero probability of disease extinction. Direct transmission depends on one successful transmission from one host to another, whereas indirect transmission hinges on two successful transmissions—one from the original host to fomite and then back from fomite to another host upon survival. To inspect the potential impact of stochasticity associated with these different transmission pathways on the invasion potential, we performed a stochastic simulation using the Modified Poisson Tau-Leap algorithm (Cao et al. 2005). The technique has been explained in “Appendix D” and parameter values have also been presented. To understand the invasion potential, we simulated our model for a duration of 1 year for $T_H=R_{0H}+R_{0F}=1.1, 1.2$, where both $R_{0H}$ and $R_{0F}$ vary from $0\%$ to $100\%$ of $T_H$ to maintain the specified value of $T_H$. We ran 1000 simulations for each case. Among the 1000 simulations, the fraction of number of times infectious individuals, *A*(*t*) that reaches zero provides us an approximate extinction probability, which is plotted in Fig. 4. The figure shows that when $T_H=1.1$, $R_{0H}\le 0.88$ and $R_{0F}\ge 0.22$, the disease goes extinct by the end of 1 year. In contrast, when $R_{0H}>0.88$ and $R_{0F}<0.22$, the extinction probability decreases to approximately 0.9 for both cases I ($g_1(F)$) & case II ($g_2(F)$). In contrast, when $T_H=1.2$, $R_{0H}\le 0.84$ and $R_{0F}\ge 0.36$, the disease goes extinct by the end of 1 year. However, when $R_{0H}>0.84$ and $R_{0F}<0.36$, the extinction probability decreases to approximately 0.86 for both cases I and II. Therefore, the extinction probability decreases with increasing $T_H$ and indirect transmission has a higher chance of extinction compared to direct transmission.

### Vaccination Threshold for Herd Immunity

In transmissible diseases, viral pathogens in existing hosts attempt to find another host to survive, proliferate and complete the cycle of transmission. At this stage, should the susceptible hosts become significantly scarce, which means that the virus is unable to find a suitable host for transmission, the transmission cycle breaks and the virus goes extinct. This is possible if the overall population has a sufficient number of immune individuals, which is defined as the state of herd immunity (Rasmussen 2020; McDermott 2021). It is a dynamic threshold that depends on the reproduction number and, consequently, on the disease transmission rate. In our present problem, this threshold is $v_c=1-\frac{1}{T_H}$, i.e., if $v_c$ fraction of host becomes immune to the virus by vaccination or recovering from the infection, the pandemic will end. From the expression of $v_c$, it is comprehensible that if the reproduction number increases (for instance, as a consequence of increasing the transmission rate), the herd immunity threshold increases as well. Therefore, it is not rational to define a rigid threshold $v_c$ that is less than unity, and thus remove all preventive measures.

One important information that $T_H$ provides us with is the transmission pathway specific requirement of control measures for herd immunity. In the COVID-19 case, this allows us to distinguish between the requirements of control measures against direct transmission and those against indirect transmission. We can minimize indirect transmission in $T_H$ by conforming to safety practices, such as general cleanliness, good hygiene, and disinfecting surfaces, while vaccination and different forms of quarantine measures can reduce the direct transmission in $T_H$. If we do not use any measures to prevent environmental transmission, the vaccination requirement for herd immunity, $v_c$, would be $v_{c,\mathrm{max}}=1-\frac{1}{R_{0H}+R_{0F}}$. Further, if we take measures for reducing the environmental transmission, the threshold ($v_c$) would then satisfy the inequality $1-\frac{1}{R_{0H}+R_{0F}}>v_c>1-\frac{1}{R_{0H}}$. Provided that the environmental transmission could be completely stopped, the vaccination threshold would be reduced to $v_{c,\mathrm{min}}=1-\frac{1}{R_{0H}}$.

However, it is challenging to isolate the correct pathway specific transmission strength as fitting a model with non-identifiable set of parameters may induce errors of attributing the contribution of one transmission pathway to another. Therefore, we first check the identifiability of the parameters, which confirms the uniqueness of the estimated parameter values, and thus isolates the pathway specific transmissibility. To clarify this with examples, we fit our model with daily active cases of early epidemic data from Nigeria, Bangladesh, and USA (considering $g_1(F)$ as the fomite to human transmission function), and we then estimate the parameters $\beta$ and $\alpha$. The daily active cases data are taken from worldometer (https://www.worldometers.info/coronavirus/).

#### Identifiability and Fitting

Let us assume, $X=(S,A,I,R,F)$ and denote the right side of the system (1) by $\mathbb {F}$. Further, $\mathbb {P}=(\beta ,\alpha )$ be the vector of parameters to be estimated. We assume the remaining parameters to be known and summarize in Table 1 with proper citation. Here, $I(t,\mathbb {P})$ is the vector of observable and $i(t,\mathbb {P})$ is the observed data at $t=1,2,\ldots ,40$ days. We assume $i(t,\mathbb {P})$ follow Poisson distributed with mean $I(t,\mathbb {P})$, then the maximum likelihood function will be:

$$\begin{aligned} L(i(t,\mathbb {P})) \mid I(t,\mathbb {P})) =&\prod _{k=1}^{40} \dfrac{I(t_k)^{i(t_{k})} e^{-I(t_k)}}{i(t_{k})!}. \end{aligned}$$

**Table 1:** Values of the model parameters corresponding to the COVID-19 cases in USA, Bangladesh, and Nigeria

Parameter	Value (day$^{-1}$)	References
*USA*		
$\Lambda$	11, 527.9	Assuming 328.2 million population at the beginning
$\mu$	$3.5125e{-}05$	Assuming 78 year mean life time
$\omega$	$\dfrac{1}{5.2}$	Okuonghae and Omame (2020)
$\beta$	${-}$ ${-}$	Estimated(fitting)
$\rho \pi$	${-}$ ${-}$	Estimated(fitting)
$\gamma _A$	$\dfrac{1}{10}$	Adewole et al. (2021)
$\gamma _I$	$\dfrac{1}{14}$	Masud et al. (2020)
$\delta$	0.03	Assumed
$\epsilon$	2.3	Yang and Wang (2020)
$\xi$	1	Yang and Wang (2020)
*Bangladesh*		
$\Lambda$	6088.3	Assuming 160 million population at the beginning
$\mu$	$3.8052e-05$	Assuming 72 year mean life time
$\omega$	$\dfrac{1}{5.2}$	Okuonghae and Omame (2020)
$\beta$	${-}$ ${-}$	Estimated (fitting)
$\rho \pi$	${-}$ ${-}$	Estimated (fitting)
$\gamma _A$	$\dfrac{1}{10}$	Adewole et al. (2021)
$\gamma _I$	$\dfrac{1}{14}$	Garba et al. (2020)
$\delta$	0.0039	Masud et al. (2020)
$\epsilon$	3	Assumed
$\xi$	1	Yang and Wang (2020)
*Nigeria*		
$\Lambda$	10197.8	Assuming 201 million population at the beginning
$\mu$	$5.0736e-05$	Assuming 54 year mean lifetime
$\omega$	$\dfrac{1}{5.2}$	Okuonghae and Omame (2020)
$\beta$	${-}$ ${-}$	Estimated (fitting)
$\rho \pi$	${-}$ ${-}$	Estimated (fitting)
$\gamma _A$	$\dfrac{1}{10}$	Adewole et al. (2021)
$\gamma _I$	$\dfrac{1}{14}$	Garba et al. (2020)
$\delta$	0.054	Garba et al. (2020)
$\epsilon$	4	Assumed
$\xi$	1	Yang and Wang (2020)

As $\ln$ is a monotonically increasing function, we minimize the negative log likelihood function (NLF) instead of maximizing the likelihood function for computational convenience. The NLF is reduced to:

$$\begin{aligned} \text{ NLF } =&-\sum _{k=1}^{40} i(t_{k}) \ln \big (I(t_{k})\big ) +\sum _{k=1}^{40} I(t_{k}) + \sum _{k=1}^{40} \ln (i(t_{k})!). \end{aligned}$$

As the last term in the above equation remains unchanged, it is sufficient to minimize the sum of the first two terms. Therefore, the fitting process reduces to a minimization problem as,

$$\begin{aligned} \min (\text{ NLF}) = \min \left( -\sum _{k=1}^{40} i(t_{k}) \ln \big (I(t_{k})\big ) +\sum _{k=1}^{40} I(t_{k})\right) \end{aligned}$$

subject to

3 $$\begin{aligned} \begin{aligned} \dfrac{\mathrm{d}}{\mathrm{d}t} X(t,\mathbb {P})&=\mathbb {F}(X,\mathbb {P},t)\\ I(0)=I_0\\ X(t), \mathbb {P}&\ge 0 \end{aligned} \end{aligned}$$

The above fitting problem will provide practically feasible and unique parameters values if $\mathbb {P}$ is identifiable. The parameters $\mathbb {P}$ is structurally identifiable if a unique solution $X(t,\mathbb {P})$ exists for each $\mathbb {P}$ and a fixed initial condition. First, we estimate the fisher information matrix (*FIM*) and then compute the profile likelihoods to confirm the identifiability of the parameters.

We have observations at 40 distinct times, a system of 5-state variables, and two unknown parameters. Therefore, the sensitivity matrix *M* consists of 5 time-dependent $5\times 2$ blocks $\mathbb {A}(t_k)$

$$\begin{aligned} M=\begin{bmatrix} \mathbb {A}(t_1)\\ \mathbb {A}(t_2)\\ \vdots \\ \ \mathbb {A}(t_5)\\ \end{bmatrix} \end{aligned}$$

where $$\mathbb {A}_{jn}(t_k)= \dfrac{\partial x_j(t_k,\mathbb {P})}{\partial P_n}, \quad k=1,\ldots , 40, \ n=1,2 \text{ and } j=1,\ldots ,5.$$

The $2\times 2$ FIM is $FIM=M^T M$, which has 2 columns. Let us denote the parameter estimates as $\hat{\beta }$ and $\hat{\alpha }$. We approximate the FIM numerically by perturbing $\hat{\beta }$ to the values $\hat{\beta ^+}=(1+0.001)\hat{\beta }$ and $\hat{\beta ^-}=(1-0.001)\hat{\beta }$, for which we integrate the model for each observation time. Then, we approximate the derivatives, $\mathbb {A}_{j1}(t_k)=\dfrac{\partial x_j(t_k,\hat{\beta },\hat{\alpha })}{\partial \hat{\beta }}, \ k=1,\ldots ,40, \ j=1,\ldots 5$ numerically, whereas $\hat{\alpha }$ remain fixed. This provides the first column. We repeat the same process for $\hat{\alpha }$ to obtain the second column. Then, we check the rank of the matrix *FIM*, which is 2, which ensures that the parameters have no implicit dependency. This confirms the structural identifiability numerically. Further. we investigate practical identifiability to confirm whether the parameters estimated by fitting this model with this set of data are capable of differentiating the role of the different transmission pathways.

To investigate practical identifiability, we compute the profile likelihood of the parameters ${\beta }$ and ${\alpha }$. Profile likelihood reveals the dependency of the *NLF* on each parameter, and exposes the minimization of the *NLF* at the estimated value. The desired profile likelihoods are as follows:

$$\begin{aligned} PL_{\beta }(\beta )= \min \limits _{\alpha }\big \{ NLF(\beta ,\alpha ) \big \} \text{ and } PL_{\alpha }(\alpha )= \min \limits _{\beta }\big \{ NLF(\beta ,\alpha ) \big \} \end{aligned}$$

where $$\beta \in [\hat{\beta }(1-0.05), \hat{\beta }(1+0.05)]$$ and $$\alpha \in [\hat{\alpha }(1-0.05), \hat{\alpha }(1+0.05)]$$.

**Fig. 5:**
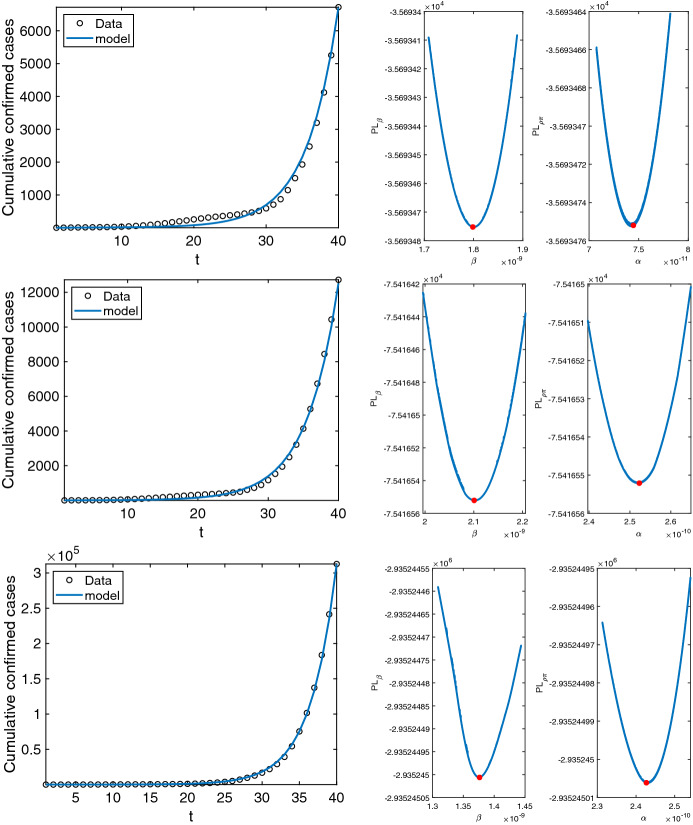
Data fitting with Likelihood profile. Graphs showing data fitting and likelihood profiles of the estimated parameters for the early days of COVID-19 outbreak in Nigeria, Bangladesh, and USA, respectively (color figure online)

Figure 5 shows the fitting along with the profile likelihood of the parameters which shows unique minima of the NLF at the estimated value of the parameters (second and third column) and hence confirms the identifiability which informs pathway specific transmission potential. The corresponding estimates of the reproduction numbers along with bounds for vaccination threshold are shown in Table 2 for each of the three countries.

### Role of Environmental Transmission

**Fig. 6:**
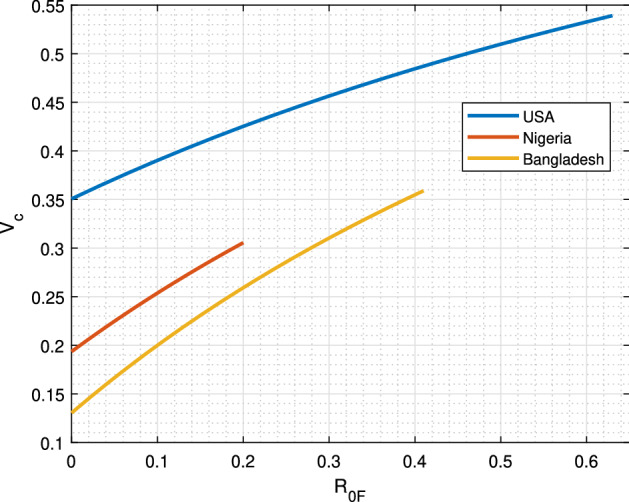
Estimated vaccination threshold. Graph showing the vaccination thresholds for USA, Nigeria, and Bangladesh as a function of $R_{0F}$. Early COVID-19 data from Nigeria, Bangladesh, and USA were used for the estimation. Data are available online on Worldometer at https://www.worldometers.info. The estimated vaccination thresholds for these countries are 0.539, 0.306, and 0.360, respectively. However, these could be further decreased to a minimum of 0.353, 0.191, and 0.130, respectively, by reducing $R_{0F}$, i.e., preventing indirect transmission (color figure online)

Figure 6 depicts the vaccination thresholds for these three different countries as a function of $R_{0F}$, and it clearly shows that the vaccination threshold would be decreased to a minimum value $v_{c,\mathrm{min}}=1-\frac{1}{R_{0H}}$ when $R_{0F}=0$, i.e., the vaccination requirement would reach its minimum value $v_{c,\mathrm{min}}$, which is the y-intercept, if we manage to take sufficient measures to ensure no environmental/indirect transmission. In contrast, when the environmental transmission is partially halted, or if no measures are taken whatsoever, the vaccination threshold would be increased to a maximum value of $v_{c,\mathrm{max}}=1-\frac{1}{R_{0H}+R_{0F}}$ depending on the size of $R_{0F}$. Moreover, as the indirect transmission possess higher chance of extinction than direct transmission, the additional indirect transmission would not increase the probability of invasion. According to our estimation from early epidemic data, provided that we limit indirect transmission, the vaccination threshold can be reduced to a minimum of 0.191, 0.130, and 0.353, in the cases of Nigeria, Bangladesh, and USA, respectively. Table 1 summarizes other parameter values used in the simulations for these three countries. It is noteworthy that as time will pass by, the estimate of both $T_H$ and $v_c$ will change.

**Table 2:** Country wise estimated rates of invasion and vaccination thresholds (approx.)

Country	$R_{0H}$	$R_{0F}$	$T_H$	$v_{c,\mathrm{min}}$	$v_{c,\mathrm{max}}$
Nigeria	1.24	0.20	1.44	0.191	0.306
Bangladesh	1.15	0.41	1.56	0.130	0.360
USA	1.54	0.63	2.17	0.353	0.539

## Discussion and Conclusion

SARS-CoV-2 can survive on different types of surfaces and has the potential to be transmitted to susceptible individuals. Therefore, our model has considered two types of transmission routes: human-to-human (direct transmission) and human to environmental fomites and then back to human (indirect transmission). Both transmission routes contribute to the reproduction number, and the degree of this epidemic is enhanced when the sum of the contribution of both direct and indirect transmissions in the type reproduction number exceeds one. The deterministic result shows that the invasion threshold is equally sensitive to both transmission routes. However, the stochastic simulation reveals that it is the indirect transmission that has a lower invasion potential compared to direct transmission.

Our analysis demonstrates that to develop effective control strategies, it is important to differentiate the role of these two different routes. The explicit modeling of both transmission routes and the estimation of the associated reproduction rates can allow us to gain a greater understanding of the indirect transmission epidemic potential and extent of efforts and measures that should be implemented in terms of disinfecting our proximal environment and maintaining personal hygiene. The analysis shows that the epidemic may persist even if direct transmission is reduced to 0 (for example, by social distancing and/or vaccination), whereas the reproduction number due to indirect transmission is $>1$ (for example, due to lack of personal hygiene). Similarly, the epidemic may persist even if the indirect transmission is reduced to 0, whereas the reproduction number due to the direct transmission is $>1$. It should be noted that it might not be practically feasible to reduce transmission from either routes to 0. If the reproduction number due to the direct transmission is $<1$ but the type reproduction number is $>1$, the epidemic could simply be terminated by maintaining strict cleanliness only. Moreover, the environmental transmission has lower invasion potential then the direct transmission. This nourishes the conclusion that, once the strength of the environmental transmission is known, direct transmission can be contained by using focused controls, such as the vaccination and/or different forms of quarantine.

Having obtained the sensitive quantification of the epidemic potential of the environmentally mediated transmission, mitigating environmental transmission by cleanliness, personal hygiene, and disinfection of the contaminated surfaces would reduce the requirement of human oriented control efforts. Note that individuals with either vaccine or acquired immunity may not be responsible for direct transmission but may play a plausible role in indirect transmission by acting as carrier. Besides, the herd immunity threshold is not a steady state; instead, it may increase due to the increasing transmission rate or increase in susceptible individuals due to loss of immunity. Therefore, having a fraction of individuals with immunity does not allow us to abort all preventive measures. Moreover, besides the implementation of vaccines, SARS-CoV-2 is rapidly developing mutations and it is likely that the vaccine-related antibodies may become ineffective to the new strains. We, therefore, conclude that all possible transmission routes need to be carefully considered and measured while vaccinating the population until the transmission is fully under control or declared eradicated.

Apart from fomite-mediated transmission, indirect transmission also includes transmission through aerosol particles deposited in the air by droplets, as suggested by Leung (2021), Castaño et al. (2021) and Aydogdu et al. (2021). However, aerosol mediated transmission is highly dependent on the respective air circulation and the physical structure of the venue. The deposition and decay rates can vary significantly. Furthermore, consideration of both indirect routes would lead to difficulties in terms of estimating the parameters associated with them. Despite this limitation, our study provides a clear indication that all possible transmission routes need to be carefully considered, and their transmission potential needs to be accurately quantified to measure the required accurate threshold for achieving herd immunity. Lastly, in estimating herd immunity and planning control strategies, immunity loss and the probability of reinfection should be considered as well, two issues that form the core of our future research studies.

## Data Availability

Data have been taken from worldometer (https://www.worldometers.info/coronavirus/).

## Appendix A: Next Generation Matrix

To find the basic reproduction number, we follow the next generation method (van den Driessche and Watmough 2002) and system (1) is written in the following form:

A1 $$\begin{aligned} \dot{x}=\mathcal {F}(x)-(\mathcal {V}^-(x)-\mathcal {V}^+(x)) \end{aligned}$$

where $\mathcal {F}$, $\mathcal {V}^-$, and $\mathcal {V}^+$ are as follows:

$$\begin{aligned}&\mathcal {F} = \left( \begin{array}{c} \beta A S + \rho S f_i(F)\\ \epsilon A\\ 0 \\ 0\\ 0\\ \end{array} \right) , \mathcal {V}^- = \left( \begin{array}{c} k_A A\\ \xi V \\ k_I I \\ \mu R\\ \beta A S + \rho S f_i(F) + \mu S\\ \end{array} \right) , \\&\mathcal {V}^+ = \left( \begin{array}{c} 0\\ 0\\ \omega A \\ \gamma _A A + \gamma _I I \\ \Lambda \\ \end{array} \right) \end{aligned}$$

and $x=(A,F,I,R,S)\in \mathbb {R}^+_5$. Here, the rate of appearance of new infections is represented by the elements of $\mathcal {F}$ and the rate of transfer of individuals into and out of the compartments are in $\mathcal {V}^+$ and $\mathcal {V}^-$, respectively. The Jacobian of $\mathcal {F}$ at $\mathcal {E}_0$ takes the following form.

$$\begin{aligned} Jacobian(\mathcal {F})_{\mathcal {E}_0} = \left( \begin{array}{cc|ccc} \beta \frac{\Lambda }{\mu } &{}\rho \frac{\Lambda }{\mu } f_i'(F) &{} 0 &{} 0 &{} 0\\ \epsilon A &{} 0 &{} 0 &{} 0 &{} 0\\ \hline 0 &{} 0 &{} 0 &{} 0 &{} 0 \\ 0 &{} 0 &{} 0 &{} 0 &{} 0\\ 0 &{} 0 &{} 0 &{} 0 &{} 0\\ \end{array} \right) \end{aligned}$$

As only the first $2\times 2$ block of the above matrix is nonzero, whatever multiplied with it would result in a similar matrix with only a first $2\times 2$ nonzero block. So, it suffices to consider a $2\times 2$ matrix instead of a $5\times 5$ matrix. Following van den Driessche and Watmough (2002), we write the sub-matrices $\mathfrak {F}$ and $\mathfrak {V}$ as

$$\begin{aligned} \mathfrak {F}=\left( \frac{\partial \mathcal {F}_i}{\partial x_j}\right) _{\mathcal {E}_0,1\le k,j\le 2}= \left( \begin{array}{cc} \beta \frac{\Lambda }{\mu } &{}\rho \frac{\Lambda }{\mu } f_i'(F)\\ \epsilon &{} 0\\ \end{array} \right) , \mathfrak {V}=\left( \frac{\partial \mathcal {V}_i}{\partial x_j}\right) _{\mathcal {E}_0,1\le i,j\le 3}= \left( \begin{array}{cc} k_A&{} 0 \\ 0 &{} \xi \\ \end{array} \right) \end{aligned}$$

## Appendix B: Proof to Theorem 1

### Proof

Conferring the symbols from equation (A1), we verify the following at $\mathcal {E}_0$.

- If $x\ge 0$, then $\mathcal {F}_i,\mathcal {F}^-_i,\mathcal {V}^+_i\ge 0$ for $i=1,2,\ldots ,5.$
- If $$x_i=0$$, then $$\mathcal {V}^-_i=0$$.
- $$\mathcal {F}_i=0$$ if $$i>2$$.
- At $$\mathcal {E}_0$$, $$\mathcal {F}_i(x)=0$$ and $$\mathcal {V}^+_i(x)=0$$.
- Finally, all eigenvalues of $\mathfrak {V}_{\mathcal {E}_0}$ are positive.

Therefore, theorem 2 from van den Driessche and Watmough (2002) completes the proof. $\square$

## Appendix C: Proof to Theorem 2

### Proof

For computational convenience, we assume $k_A$ as the bifurcation parameter. At $R_0=1$,

$$\begin{aligned} k_A^*=\frac{\Lambda \beta }{\mu }+\frac{\Lambda \epsilon \pi \rho }{\mu \xi } \end{aligned}$$

The largest eigenvalue of the Jacobian of the system at DFE and $k_A^*$ is 0 with multiplicity one. In addition, there is a nonnegative left eigenvector and a nonnegative right eigenvector corresponding to the 0 eigenvalue. The local dynamics are then determined by the two constants *a* and *b* defined in Theorem 4.1 of Castillo-Chavez and Song (2004). For our model, $a=-\frac{\beta }{2}$ and $b=\frac{\xi ^2\mu }{\Lambda \epsilon \pi \rho } k_A^{*2}$, which indicates that a positive EE exists and it is locally asymptotically stable for $R_0>1$. $\square$

## Appendix D: Modified Poisson Tau-Leap Algorithm

The model (1) can be split into the following 12 reactions:

D2 $$\begin{aligned} R_1&:&\xrightarrow {K_1} S \nonumber \\ R_2&:&S\xrightarrow {K_2} \nonumber \\ R_3&:&S\xrightarrow {K_3(Direct Transmission)} A\nonumber \\ R_4&:&S\xrightarrow {K_4((Indirect Transmission))} A\nonumber \\ R_5&:&A\xrightarrow {K_5} \nonumber \\ R_6&:&A\xrightarrow {K_6} R\nonumber \\ R_7&:&A\xrightarrow {K_7} I \nonumber \\ R_8&:&I\xrightarrow {K_8} \nonumber \\ R_9&:&I\xrightarrow {K_9} R\nonumber \\ R_{10}&:&R\xrightarrow {K_{10}}\nonumber \\ R_{11}&:&\xrightarrow {K_{11}}F\nonumber \\ R_{12}&:&F\xrightarrow {K_{12}} \end{aligned}$$

and the corresponding propensities are as follows: $a_1=\Lambda , a_2= \mu S(t), a_3=\beta A(t) S(t), a_4=S(t) g_i(F(t)), a_5=\mu A(t), a_6= \gamma _A A(t), a_7=\omega A(t), a_8= (\mu + \delta ) I(t), a_9=\gamma _I I(t), a_{10}= \mu R(t), a_{11}= \epsilon A(t)$, and $a_{12}= \xi F(t),$ respectively. The stoichiometry matrix is provided by the following matrix:

D3 $$\begin{aligned} v=\left( \begin{array}{c} v_1\\ v_2\\ \vdots \\ v_{12} \end{array}\right) =\left( \begin{array}{ccccccccc} 1 &{}&{} 0 &{}&{} 0 &{}&{} 0 &{}&{} 0 \\ -1 &{}&{} 0 &{}&{} 0 &{}&{} 0 &{}&{} 0 \\ -1 &{}&{} 1 &{}&{} 0 &{}&{} 0 &{}&{} 0 \\ -1 &{}&{} 1 &{}&{} 0 &{}&{} 0 &{}&{} 0 \\ 0 &{}&{}-1 &{}&{} 0 &{}&{} 0 &{}&{} 0 \\ 0 &{}&{} -1 &{}&{} 0 &{}&{}1 &{}&{} 0 \\ 0 &{}&{} -1&{}&{} 1 &{}&{} 0 &{}&{} 0 \\ 0 &{}&{} 0 &{}&{} -1&{}&{} 0 &{}&{} 0 \\ 0 &{}&{} 0 &{}&{} -1&{}&{} 1 &{}&{} 0 \\ 0 &{}&{} 0 &{}&{} 0 &{}&{}-1 &{}&{} 0 \\ 0 &{}&{} 0 &{}&{} 0 &{}&{} 0 &{}&{} 1 \\ 0 &{}&{} 0 &{}&{} 0 &{}&{} 0 &{}&{}-1 \end{array} \right) \end{aligned}$$

The propensity vector $a=[a_1(X),a_2(X),\ldots ,a_{12}(X)]^T$ along with the state change vectors $v_1,v_2,\ldots ,v_{12}$ describe the reactions $R_1,R_2,\ldots ,R_{12}$,respectively.

$$\begin{aligned}&\nabla a :=\left( \begin{array}{c} \nabla a_1\\ \nabla a_2\\ \vdots \\ \nabla a_{12} \end{array}\right) = \left( \begin{array}{ccccccccc} 0 &{}&{} 0&{}&{} 0 &{}&{} 0 &{}&{} 0\\ \mu &{}&{} 0&{}&{} 0 &{}&{} 0 &{}&{} 0\\ \beta A(t) &{}&{} \beta S(t) &{}&{} 0 &{}&{} 0 &{}&{} 0\\ g_i(F(tt)) &{}&{} 0 &{}&{} 0 &{}&{} 0 &{}&{} \frac{\partial g_i(F)t))}{\partial F} S(t)\\ 0 &{}&{} \mu &{}&{} 0 &{}&{} 0 &{}&{} 0\\ 0 &{}&{} \gamma _A &{}&{} 0 &{}&{} 0 &{}&{} 0\\ 0 &{}&{} \omega &{}&{} 0 &{}&{} 0 &{}&{} 0\\ 0 &{}&{} 0&{}&{} \mu +\delta &{}&{} 0 &{}&{} 0\\ 0 &{}&{} 0&{}&{} \gamma _I &{}&{} 0 &{}&{} 0\\ 0 &{}&{} 0&{}&{} 0 &{}&{} \mu &{}&{} 0\\ 0 &{}&{} \epsilon &{}&{} 0 &{}&{} 0&{}&{} 0\\ 0 &{}&{} 0&{}&{} 0 &{}&{} 0 &{}&{} \xi \end{array}\right) \\&\psi = \nabla a*v^T*a, \sigma ^2 = (\nabla a*v^T)^2*a \end{aligned}$$

Here, $(.)^2$ stands for the element-wise square and $*$ stands for the matrix product. Therefore, following Cao et al. (2005) we define

$$\begin{aligned} \tau :=\min _{j\in \{1,2,3,\ldots ,12\}}\left( \frac{\varepsilon a_0}{|\psi _j|},\frac{\varepsilon ^2a_0^2}{\sigma ^2_j}\right) . \end{aligned}$$

Furthermore, $L_j=\min ^{(v_{ij}<0)}_{i\in \{1,2,\ldots ,5\}}\left[ \frac{x_i}{|v_{ij}|}\right]$. Here, the square bracket represents the “greatest integer” operator. The simulation is completed according to Algorithm 1.

## Declarations

### Code Availability

Available on request.
